# Supplementary figures and images for: KIS counteracts PTBP2 and regulates alternative exon usage in neurons
Source: eLife. 2024 Apr 10;13:e96048. doi: 10.7554/eLife.96048 (PMC11045219; doi:10.7554/eLife.96048)

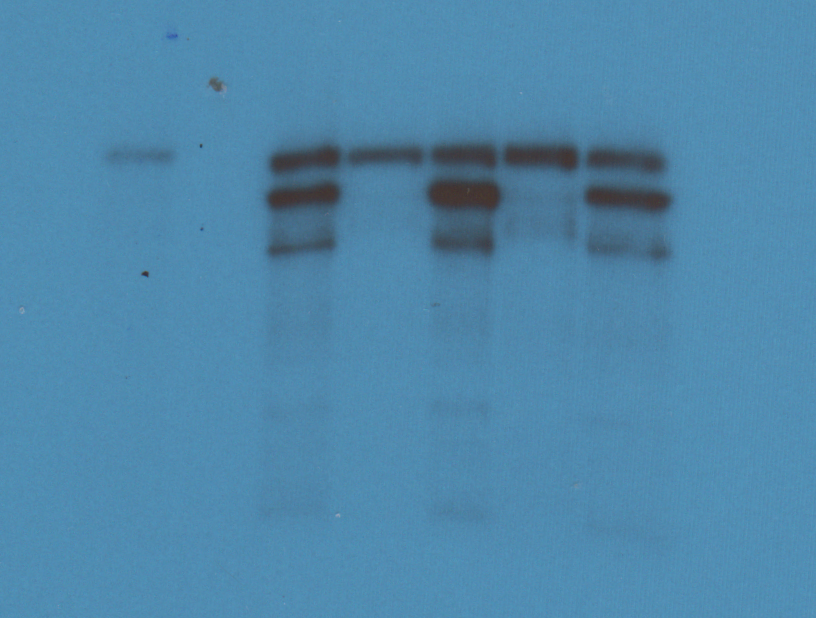

Supplement: Figure 2—figure supplement 1—source data 1. [file elife-96048-fig2-figsupp1-data1.zip › Figure 2-figure supplement 1-source data 1/32P autoradiography.tif]

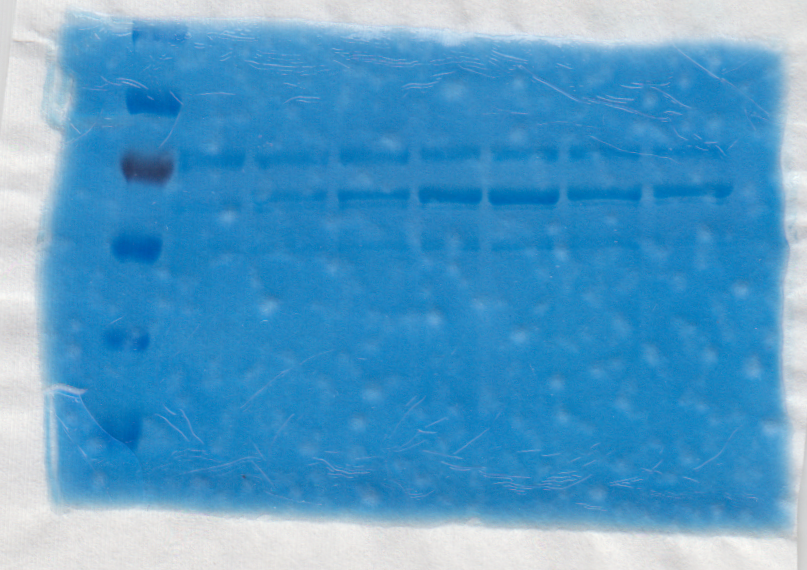

Supplement: Figure 2—figure supplement 1—source data 1. [file elife-96048-fig2-figsupp1-data1.zip › Figure 2-figure supplement 1-source data 1/Comassie Blue.tif]

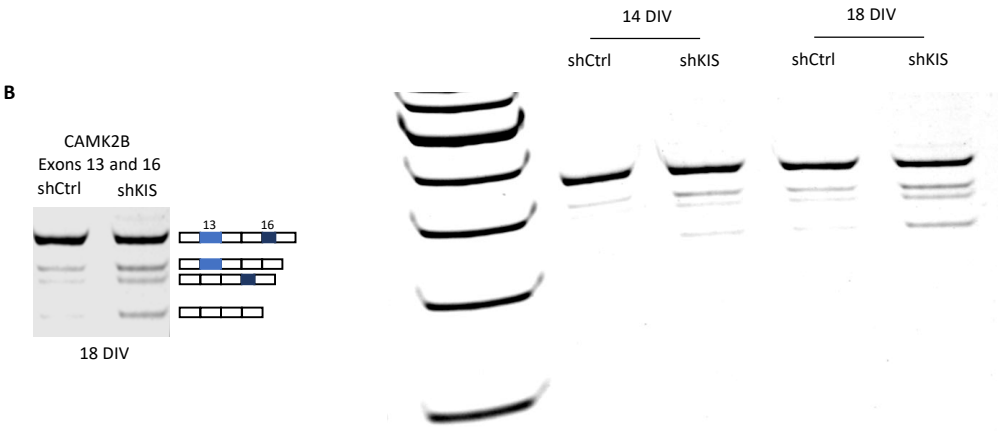

Figure 3-source data 1. CAMK2B exon exclusion analysis

Supplement: Figure 3—source data 1. [file elife-96048-fig3-data1.zip › Figure 3-source data 1/Figure 3-source data 1.pdf]

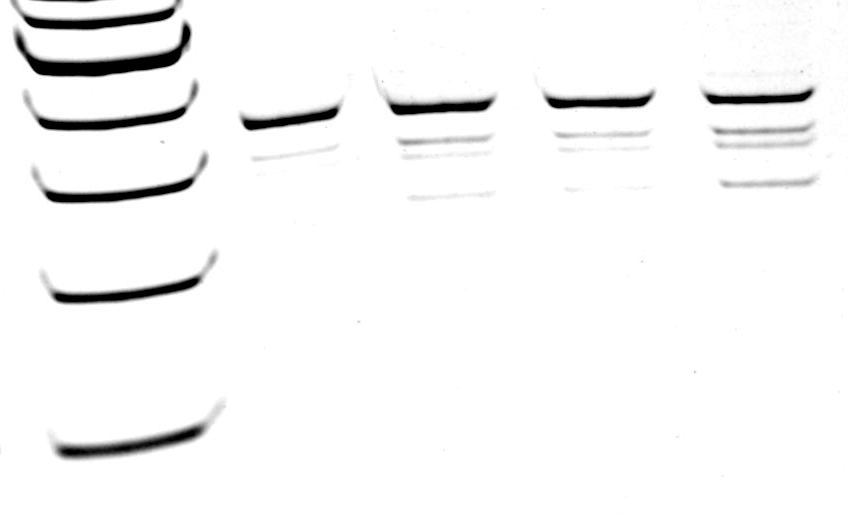

Supplement: Figure 3—source data 1. [file elife-96048-fig3-data1.zip › Figure 3-source data 1/RT-PCR.tif]

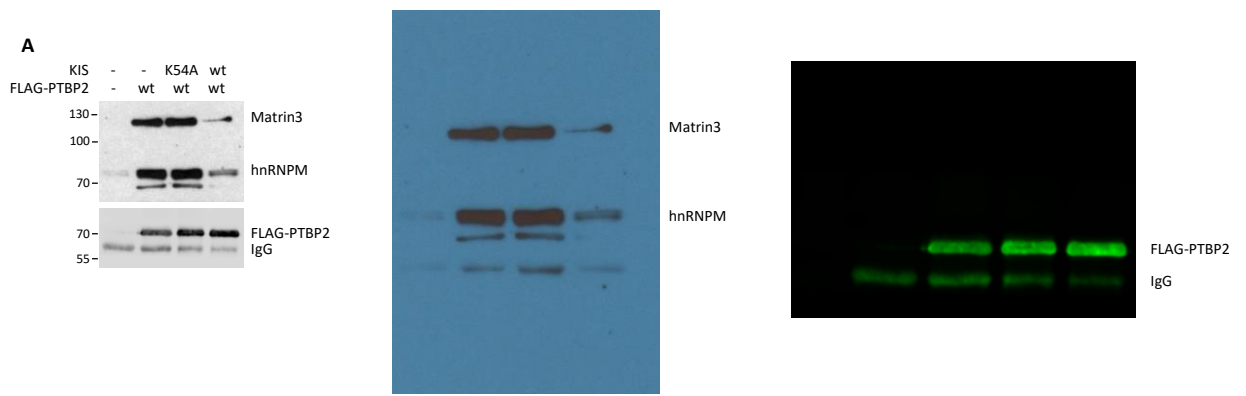

Figure 5-source data 1. hnRNPM and Matrin3 in FLAG-PTBP2 immunoprecipitates *in vivo*

Supplement: Figure 5—source data 1. [file elife-96048-fig5-data1.zip › Figure 5-source data 1/Figure 5-source data 1.pdf]

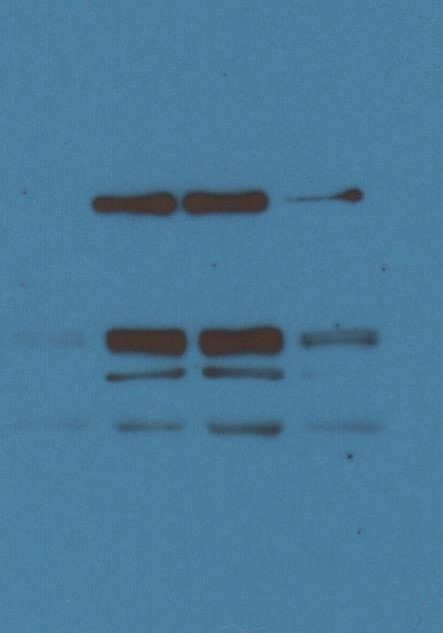

Supplement: Figure 5—source data 1. [file elife-96048-fig5-data1.zip › Figure 5-source data 1/Matrin3 and hnRNPM immunoblot.jpeg]

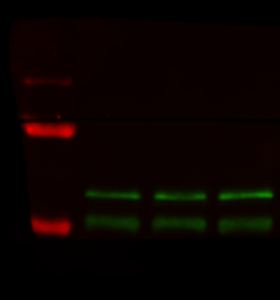

Supplement: Figure 5—figure supplement 1—source data 1. [file elife-96048-fig5-figsupp1-data1.zip › Figure 5-figure supplement 1-source data 1/FLAG immunoblot.tif]

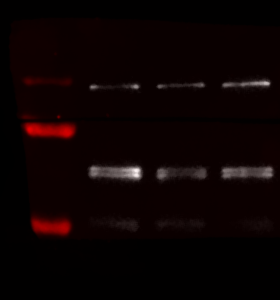

Supplement: Figure 5—figure supplement 1—source data 1. [file elife-96048-fig5-figsupp1-data1.zip › Figure 5-figure supplement 1-source data 1/Matrin3 and hnRNPM immunoblot.tif]

**C**

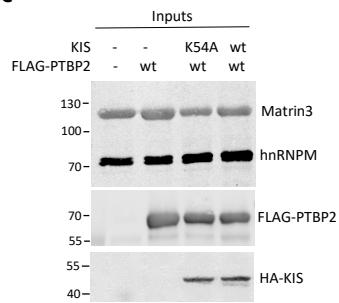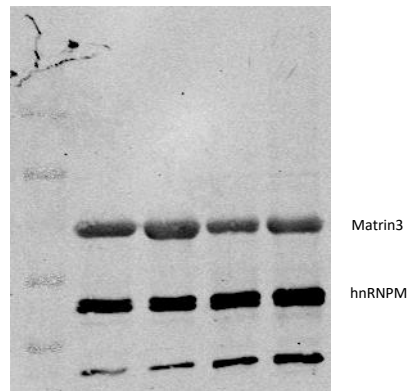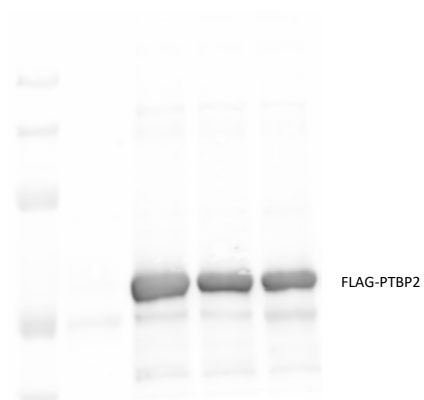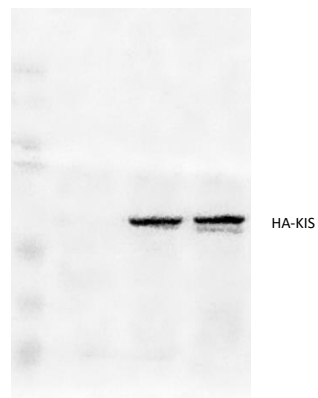

Figure 5-figure supplement 1-source data 2. Representative blot of input samples from Figure 5A

Supplement: Figure 5—figure supplement 1—source data 2. [file elife-96048-fig5-figsupp1-data2.zip › Figure 5-figure supplement 1-source data 2/Figure 5-figure supplement 1-source data 2.pdf]

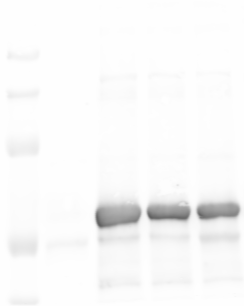

Supplement: Figure 5—figure supplement 1—source data 2. [file elife-96048-fig5-figsupp1-data2.zip › Figure 5-figure supplement 1-source data 2/FLAG-PTBP2.tif]

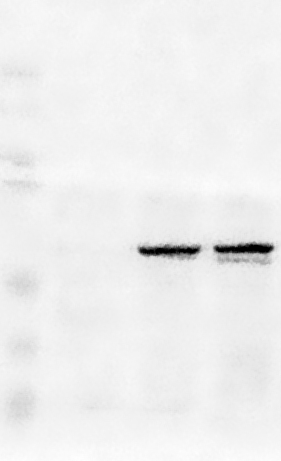

Supplement: Figure 5—figure supplement 1—source data 2. [file elife-96048-fig5-figsupp1-data2.zip › Figure 5-figure supplement 1-source data 2/KIS HA.tif]

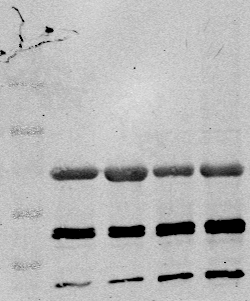

Supplement: Figure 5—figure supplement 1—source data 2. [file elife-96048-fig5-figsupp1-data2.zip › Figure 5-figure supplement 1-source data 2/Matrin3_hnRNPM.tif]

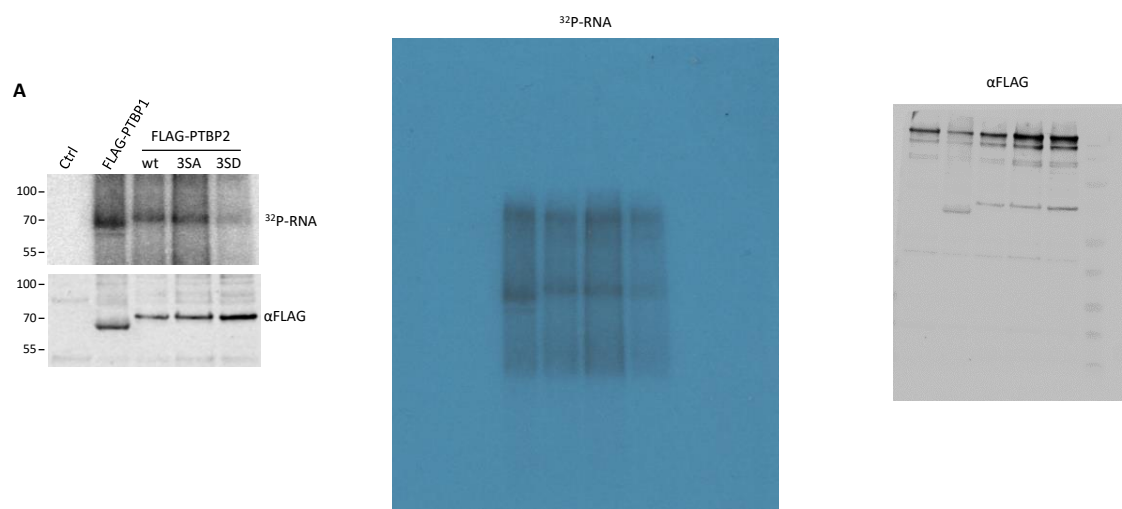

Figure 6-source data 1. PTBP2 RNA binding by CLIP

Supplement: Figure 6—source data 1. [file elife-96048-fig6-data1.zip › Figure 6-source data 1/Figure 6-source data 1.pdf]

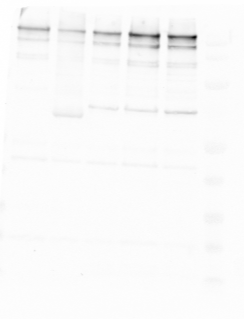

Supplement: Figure 6—source data 1. [file elife-96048-fig6-data1.zip › Figure 6-source data 1/FLAG immunoblot.tif]

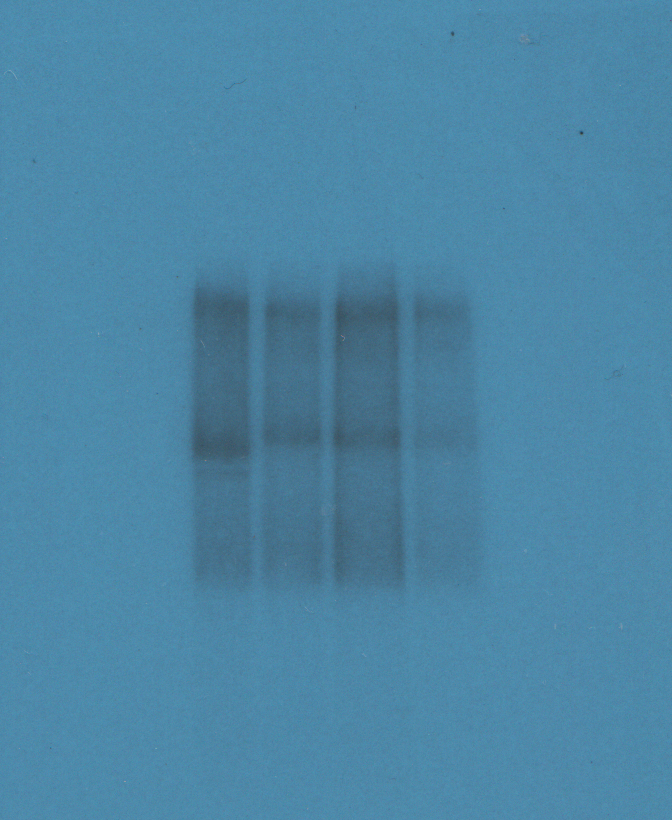

Supplement: Figure 6—source data 1. [file elife-96048-fig6-data1.zip › Figure 6-source data 1/iCLIP autoradiography.tif]

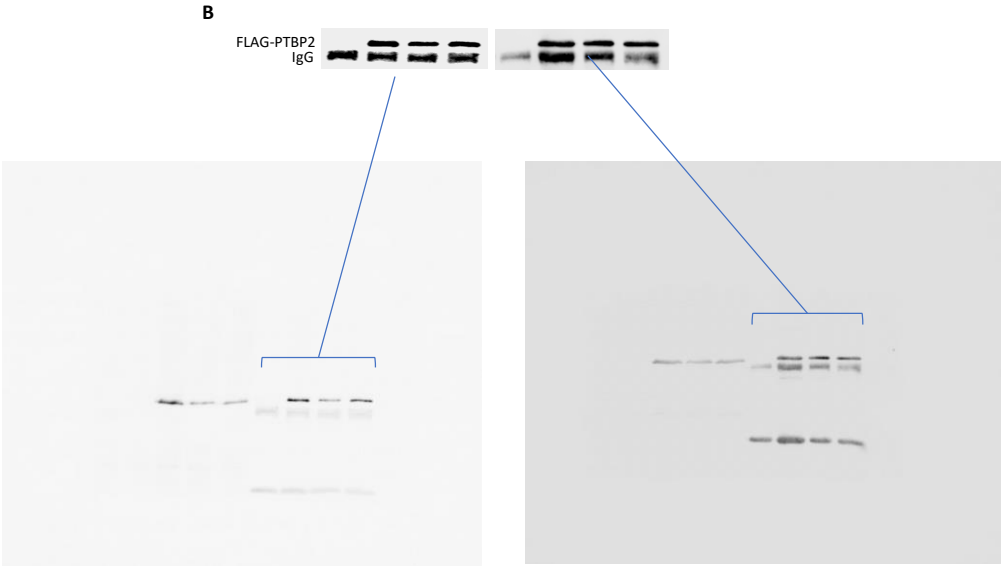

Figure 6-source data 2. FLAG-PTBP2 immunoprecipitated levels for CLIP analysis in Figure 6B

Supplement: Figure 6—source data 2. [file elife-96048-fig6-data2.zip › Figure 6-source data 2/Figure 6-source data 2.pdf]

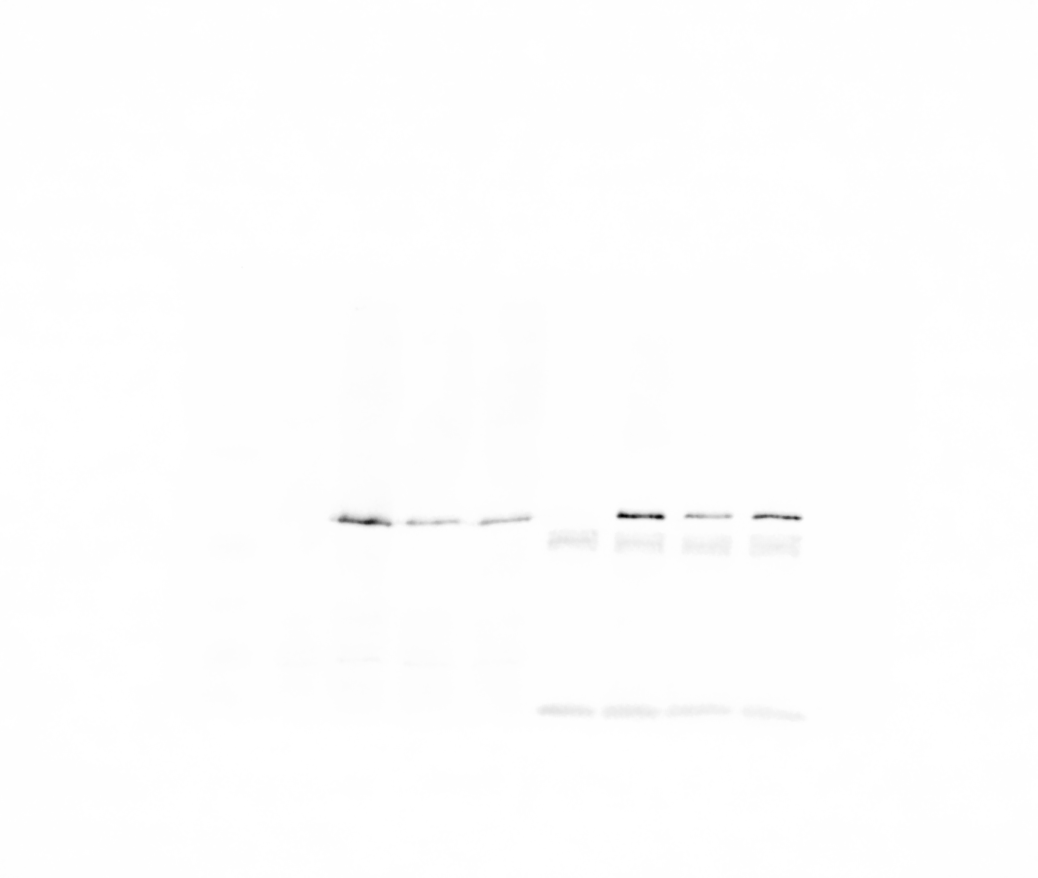

Supplement: Figure 6—source data 2. [file elife-96048-fig6-data2.zip › Figure 6-source data 2/FLAG immunoblot (left).tif]

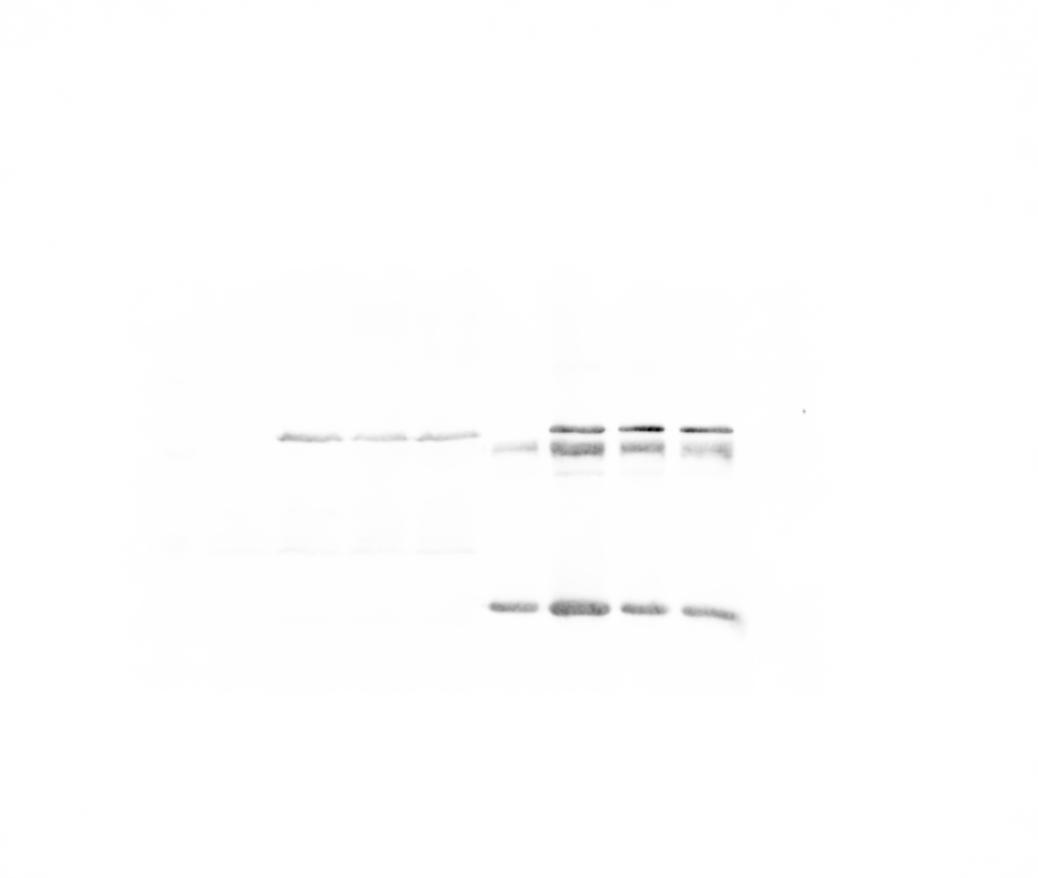

Supplement: Figure 6—source data 2. [file elife-96048-fig6-data2.zip › Figure 6-source data 2/FLAG immunoblot (right).tif]
