# Supplementary material for: KIS counteracts PTBP2 and regulates alternative exon usage in neurons
Source: eLife. 2024 Apr 10;13:e96048. doi: 10.7554/eLife.96048 (PMC11045219; doi:10.7554/eLife.96048)
Supplement: Figure 5—figure supplement 1—source data 1. [file elife-96048-fig5-figsupp1-data1.zip › Figure 5-figure supplement 1-source data 1/Figure 5-figure supplement 1-source data 1.pdf]

**A**

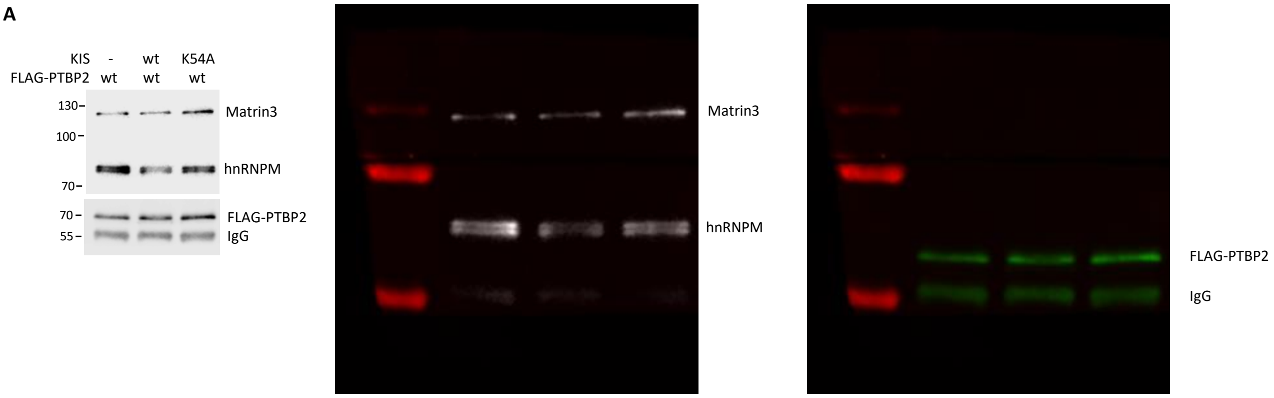


Figure 5-figure supplement 1-source data 1. hnRNPM and Matrin3 in FLAG-PTBP2 immunoprecipitates *in vitro*
